# A new basic probability assignment generation and combination method for conflict data fusion in the evidence theory

**DOI:** 10.1038/s41598-023-35195-4

**Published:** 2023-05-25

**Authors:** Yongchuan Tang, Yonghao Zhou, Xiangxuan Ren, Yufei Sun, Yubo Huang, Deyun Zhou

**Affiliations:** 1 School of Microelectronics, Northwestern Polytechnical University, Xi’an, 710072 Shaanxi China; 2 School of Computer Science, Northwestern Polytechnical University, Xi’an, 710072 Shaanxi China; 3 Hongshen Honors School, Chongqing University, Chongqing, 401331 China; 4 School of Engineering, University of Warwick, Coventry, CV4 7AL UK

**Keywords:** Computer science, Information technology, Information theory and computation

## Abstract

Dempster–Shafer evidence theory is an effective method to deal with information fusion. However, how to deal with the fusion paradoxes while using the Dempster’s combination rule is still an open issue. To address this issue, a new basic probability assignment (BPA) generation method based on the cosine similarity and the belief entropy was proposed in this paper. Firstly, Mahalanobis distance was used to measure the similarity between the test sample and BPA of each focal element in the frame of discernment. Then, cosine similarity and belief entropy were used respectively to measure the reliability and uncertainty of each BPA to make adjustments and generate a standard BPA. Finally, Dempster’s combination rule was used for the fusion of new BPAs. Numerical examples were used to prove the effectiveness of the proposed method in solving the classical fusion paradoxes. Besides, the accuracy rates of the classification experiments on datasets were also calculated to verify the rationality and efficiency of the proposed method.

## Introduction

Information fusion refers to the process of integrating and analyzing the observed data from multiple sensors to make accurate evaluations and decisions^1–3^. This technology has been developed since the research on sonar signal processing system funded by the U.S. Department of Defense in the 1970s and was applied in the field of air combat at first. It is mainly proposed for the data fusion of radar, infrared and other multi-source sensors to improve the assessment of air combat situation. In the era of big data, information fusion can be found in various industries. For example, information fusion technology can play an important role in coal mine safety monitoring system^4^ to promote its accuracy, sensitivity and stability. Besides, it is often applied in vehicle positioning and navigation in the field of intelligent transportation^5^, realizing the enhancement of the mobility and safety of the transportation system.

Dempster–Shafer (D–S) evidence theory is an effective theoretical tool to deal with information fusion. In 1967, Dempster^6^ derived the concept of upper and lower probability in dealing with multi-valued mapping of propositions and sets, and used a probability range to represent the uncertainty of an event. The Dempster’s combination rule was proposed for combining evidence from two independent information sources for some statistical problems. In 1976, Dempster’s student Shafer^7^ published *A Mathematical Theory of Evidence*, introducing the mass function and developing a method to deal with uncertainty problems based on “evidence” and “combination”. The publication of this book also marks that the D–S evidence theory has officially become a theory to quantify and calculate the uncertainty. D–S evidence theory has also been applied in the expert system^8,9^, classification with uncertainty^10–12^, clustering^13–15^, fault diagnosis^16,17^ and many other fields^18–21^, for it does not depend on the prior probability and shows an advantage in dealing with subjective judgement^8^.

However, a counter-intuitive result may be obtained when using Dempster’s combination rule for the data with high conflict^22^. How to effectively resolve the paradoxes arising from conflicting evidence has always been a hot issue in the evidence theory. Many scholars have carried out a lot of researches on this problem. One point of view suggests that Dempster’s combination rule is inadequate and a series of new evidence combination rules was proposed^23^, while the other point of view holds that the evidence source model is defective and some methods of pre-processing the evidence source were provided^24–27^. In addition, some scholars believe that the conflict comes from the incompleteness of the frame of discernment and proposed the basic framework of the generalized evidence theory^28^.

The modification of Dempster’s combination rule was first proposed by Yager^29^, who pointed out that Dempster’s combination rule constructed a new confidence structure based on the information without conflict, while the normalization step ignored evidence contradiction, so that the results might be contrary to the common sense. He argued that when the frame of discernment was a finite set, the conflict should be removed and assigned to the whole set. The modification of Dempster’s combination rule is mainly to solve the problem of redistributing conflict. In 2002, Lefevre et al.^30^ proposed the generalized framework for the fusion of information, which can be taken as a representative of this kind of method and other modification rules can be derived from this generalized framework.

The common idea for modifying evidence sources is by using the evidence discount method, and the degree of conflict between evidence should be measured before that. Jousselme et al.^31^ proposed a distance formula to measure the conflict degree between two bodies of evidence in 2001. Liu^32^ combined the classical conflict coefficient and Pignistic probability^33^ to measure conflict in the form of binary group. Silva^34^ defined three classes of conflict to measure different levels of conflict and provided a multi-criteria analysis method. Deng et al.^35^ introduced the distance function to obtain the credibility of each evidence, which was used as the coefficient for evidence discount and data fusion. Additionally, there are also lots of research about the measure of the uncertainty of evidence for evidence evaluation and modification^36,37^. Deng entropy is also defined and extended to measure the uncertainty of evidence^38,39^. Han et al.^40^ proposed an optimization method based on the fuzzy membership function to measure the uncertainty. Besides, evidential reasoning (ER) model^41–43^ can also be used for uncertain evidence modeling and processing to address the problem for combination of evidence with high conflict.

Recently, Bi et al.^44^ enhanced evidence similarity measure based on the Tanimoto measure to modify the evidence sources based on the credibility of different evidence and the weights of conflicted parts of evidence. Li et al.^45^ proposed a new discount coefficient by Deng entropy and the entropy difference between initial body of evidence and its negation. Jing et al.^46^ proposed a new base basic probability assignment method to solve the conflict problem by assigning the base belief to basic events in the frame of discernment. Wu and Tang integrated the ambiguity measure to measure the uncertainty of subjective assessment and improve the mode and effects analysis model based on the framework of D–S evidence theory^47^.

The contribution of this paper is as follows. First, the reasons for the fusion paradoxes with high conflict data are expounded. Then, inspired by the evidence discount method, an improved method for eliminating paradoxes by integrating the cosine similarity of evidence and belief entropy to adjust the evidence sources is proposed in this paper. The approach includes two parts for the generation and combination of BPA respectively, and both the reliability and uncertainty of evidence are taken into consideration. Finally, some examples and experiments are provided to demonstrate the effectiveness, superiority and rationality of the proposed method.

This rest of this paper is organized as follows. The preliminaries, including some basic concepts and theories, are introduced in “Preliminaries”. In “A new BPA generation and combination method”, a novel generation and combination method based on modifying evidence sources is proposed. Some numerical examples are presented in “Numerical examples” to verify the effectiveness and superiority of the method in dealing with both conflicting data and non-conflicting data. “Application” presents the experiments on Iris and wine datasets. “Conclusion” is the conclusion of this paper.

## Preliminaries

### Dempster–Shafer evidence theory

#### Basic concepts

Several important concepts in evidence theory should be introduced at first, such as the frame of discernment, the mass function, focal element, the belief function (*Bel*) and the plausibility function (*Pl*).

##### Definition 1

The frame of discernment^7^ is defined as a complete set composed of *N* mutually exclusive elements, which can be expressed as:

1 $$\begin{aligned} \Theta =\{{{\Theta }_{1}},{{\Theta }_{2}},\ldots ,{{\Theta }_{N}}\} \end{aligned}$$

##### Definition 2

The mass function^7^, which is also called basic probability assignment (BPA), is a mapping: $m:{{2}^{\Theta }}\rightarrow [0,1]$, that satisfies:

2 $$\begin{aligned} m(\varnothing )=0,\sum \limits _{A\subseteq \Theta }{m(A)=1} \end{aligned}$$

where $\Theta$ is the frame of discernment, ${{2}^{\Theta }}$ is the set composed of all subsets of $\Theta$, *m*(*A*) is the mass function of *A* , namely, the reliability assigned to *A*.

##### Definition 3

Focal element^7^ refers to any subset *A* of the frame of discernment that satisfies the condition:

3 $$\begin{aligned} m(A)>0 \end{aligned}$$

##### Definition 4

The belief function^7^ $Bel:{{2}^{\Theta }}\rightarrow [0,1]$ is defined as:

4 $$\begin{aligned} Bel(A)=\sum \limits _{B\subset A}{m}(B) \end{aligned}$$

##### Definition 5

The plausibility function^7^ $Pl:{{2}^{\Theta }}\rightarrow [0,1]$ is defined as:

5 $$\begin{aligned} Pl(A)=\sum \limits _{B\cap A\ne \varnothing }{m}(B) \end{aligned}$$

For any subset *A* of the frame of discernment, *Pl*(*A*) is called the upper bound probability of the proposition *A*, which represents the potential evidence support degree for proposition *A*. The length of the belief interval [*Bel*(*A*), *Pl*(*A*)] represents the imprecision of proposition *A*.

#### Dempster’s combination rule

Mark $Be{{l}_{1}}$ and $Be{{l}_{2}}$ as the belief functions of the frame of discernment $\Theta$, and the mass functions are ${{m}_{1}}$ and ${{m}_{2}}$ respectively. ${{m}_{1}}$ and ${{m}_{2}}$ are obtained from two independent sources of evidence, and the corresponding focal elements are ${{A}_{1}},{{A}_{2}},\ldots ,{{A}_{k}}$ and ${{B}_{1}},{{B}_{2}},\ldots ,{{B}_{k}}$. When the following condition is satisfied:

6 $$\begin{aligned} \sum \limits _{{{A}_{i}}\cap {{B}_{j}}=\varnothing }{{{m}_{1}}({{A}_{i}}){{m}_{2}}({{B}_{j}})<1} \end{aligned}$$

For any subset $\text {A}\subset \Theta$:

7 $$\begin{aligned} m(\varnothing )=0,m(A)=\frac{\sum \nolimits _{{{A}_{i}}\cap {{B}_{j}}=A}{{{m}_{1}}({{A}_{i}}){{m}_{2}}({{B}_{j}})}}{1-\sum \nolimits _{{{A}_{i}}\cap {{B}_{j}}=\varnothing }{{{m}_{1}}({{A}_{i}}){{m}_{2}}({{B}_{j}})}} \end{aligned}$$

$m:{{2}^{\Theta }}\rightarrow [0,1]$, resulting from the fusion of ${{m}_{1}}$ and ${{m}_{2}}$ according to Dempster’s combination rule, can be verified as a mass function. Mark the conflict part as $k=\sum \nolimits _{{{A}_{i}}\cap {{B}_{j}}=\varnothing }{{{m}_{1}}({{A}_{i}}){{m}_{2}}({{B}_{j}})}$, and the greater the k is, the greater the conflict degree between the evidence is. The associative and commutative laws are satisfied when evidence is fused with Dempster’s combination rule:

$$\begin{aligned} ({{m}_{1}}\oplus {{m}_{2}})\oplus {{m}_{3}}={{m}_{1}}\oplus ({{m}_{2}}\oplus {{m}_{3}}) \end{aligned}$$

$$\begin{aligned} {{m}_{1}}\oplus {{m}_{2}}={{m}_{2}}\oplus {{m}_{1}} \end{aligned}$$

### High conflict data fusion problem in the classical D–S evidence theory

The classical method (DS evidence theory) cannot deal with conflicting data fusion because it usually produces counter-intuitive results. Zadeh^48^ gave an example:

Let the frame of discernment

$$\Theta =\{a,b,c\}$$

and the two BPAs are as follows:

$$m_1(\{a\}) = 0.99; m_1(\{b\}) = 0.01; m_1(\{c\}) = 0.$$

$$m_2(\{a\}) = 0; m_2(\{b\}) = 0.01; m_2(\{c\}) = 0.99.$$

According to the D–S evidence theory:

8 $$\begin{aligned} m({a}) = 0; m({b}) = 1; m({c}) = 0 \end{aligned}$$

Surprisingly, the BPA of $\{b\}$ is 1, which is obviously counter-intuitive. There are many such examples and real cases. So we should improve the classical D–S evidence theory if we want to handle high conflict evidence.

### Evidence modification and fusion methods

Some typical evidence modification and fusion methods are used for comparison with the proposed method.

#### Yager’s method

Yager^29^ proposed a new method to handle the conflict evidence. He added conflict information as unknown information to $m(\Theta )$ and therefore improved the classical combination rules as follows:

9 $$\begin{aligned} m(\emptyset )=0 \end{aligned}$$

10 $$\begin{aligned} m(A)= \left\{ \begin{array}{l}\sum _{A_i\bigcap B_i=A}m_1(A_i)m_2(B_i) \qquad A\ne \Theta \\ \\ \sum _{A_i\bigcap B_i=\Theta }m_1(A_i)m_2(B_i)+k \qquad A=\Theta \end{array}\right. \end{aligned}$$

11 $$\begin{aligned} k=\sum _{A_i\bigcap B_i=\emptyset }m_1(A_i)m_2(B_i) \end{aligned}$$

#### Murphy’s method

Murphy^49^ did not change the classical combination rules. He changed the model. Suppose there are n evidences, this method first averages the BPA of n evidences to obtain $m_{avg}$: $f_{DS}(S_1,S_2)$ represents the DS combination rules of two evidence sources. And $m^i$ represents BPA after the ith iteration. The Murphy combination rule are as follows:

12 $$\begin{aligned} m^1=f_{DS}(m_{avg},m_{avg}) \end{aligned}$$

13 $$\begin{aligned} m^i=f_{DS}(m^{i-1},m_{avg}), i\ge 2 \end{aligned}$$

#### Dubois and Prade’s method

Dubois and Prade^50^ proposed a different method to handle conflict evidence. Let us suppose the source $E_1$ supports the subset A with the mass of belief $m_1(A)$, and the source $E_2$ supports the subset B with the mass of belief $m_2(b)$. The combination rule are as follows:

14 $$\begin{aligned} m(C)=\sum _{A\bigcap B=C}m_1(A)m_2(B)+\sum _{A\bigcap B=\emptyset ,A\bigcup B=C}m_1(A)m_2(B) \end{aligned}$$

### Belief entropy

#### Definition 6

Deng entropy^38^ can be presented as follows:

15 $$\begin{aligned} {{E}_{D}}=-\sum \limits _{A\subseteq \Theta }{m(A){{\log }_{2}}\frac{m(A)}{{{2}^{|A|}}-1}} \end{aligned}$$

Where *m* is the mass function, $\Theta$ is the frame of discernment, |*A*| is the cardinality of *A*. When BPA degenerates into probability, confidence entropy degenerates into Shannon entropy.

### Mahalanobis distance (M-distance)

#### Definition 7

The M-distance between a point *x* and the population can be expressed as:

16 $$\begin{aligned} {{d}^{2}}(x,\mu )={{(x-\mu )}^{T}}{{\Sigma }^{-1}}(x-\mu ) \end{aligned}$$

Where, $\mu$ is the mean of the population and $\Sigma$ is the covariance matrix of the population. If $\mu$ and $\Sigma$ are unknown, they can be replaced by the sample mean and the sample covariance matrix.

### Interval similarity

#### Definition 8

Mark $A=[{{a}_{1}},{{a}_{2}}],B=[{{b}_{1}},{{b}_{2}}]$ as two intervals. The similarity between them can be defined as follows:

17 $$\begin{aligned} S(A,B)=\frac{1}{1+\alpha D(A,B)} \end{aligned}$$

Where, $\alpha (\alpha >0)$ is the supporting coefficient, and *D*(*A*, *B*) is the distance between interval *A* and interval *B*. The supporting coefficient $\alpha$ regulates the dispersion degree of the similarity. When interval A degenerates into a point and interval B is reduced to a countable set, *D*(*A*, *B*) and *S*(*A*, *B*) are the distance and similarity between the point and the set.

## A new BPA generation and combination method

### Explanation of the zero mass function

D–S evidence theory deals with the uncertainty when assessing and forecasting the unknown. The data and results obtained from different sensors or experts are the reflections of this uncertainty. The data and results are different because the perspectives, thinking modes and knowledge backgrounds of the sensors or experts are different. Fusion paradoxes often occur with highly contradicting evidence. It can be noticed that the paradox arises because one BPA for some focal element is assigned to 0, but other BPAs for that focal element are assigned to higher values. When it is assumed that all sensors are not faulty and all the experts are qualified, the conflict between non-zero and zero BPAs should be paid attention to. The existence of a non-zero BPA for a focal element means some corresponding evidence have been found to support it. Those zero BPAs for that focal element can be interpreted as no evidence collected or a lack of knowledge, so the zero BPAs need to be modified. Inspired by the similarity distance between the evidence^31^ and the belief entropy^38^, a new combination method based on weighted discounting of data sources was proposed with the consideration of reliability and uncertainty.

We used Mahalanobis distance to measure the similarity between the test sample and each focal element of the frame of discernment to determine BPA. Then cosine similarity and belief entropy were respectively used to measure the reliability and uncertainty of each BPA to make adjustments and generate a standard BPA. In general, we integrate the cosine similarity of evidence and belief entropy to adjust the evidence sources.

### Definition of cosine similarity

Before the introduction of the method, the definition of cosine similarity considering the relationships between focal elements needs to be explained.

#### Definition 9

Assume that ${{m}_{i}}$ and ${{m}_{j}}$ are two independent mass functions: ${{2}^{\Theta }}\rightarrow [0,1]$, and ${{X}_{i}},{{X}_{j}}$ refer to two independent evidence vectors generated by BPA on each focal elements of the frame of discernment $\Theta$. The cosine similarity between two evidence vectors can be presented in the following expression:

18 $$\begin{aligned} {{s}_{ij}}=\frac{{{X}_{i}}QX_{j}^{T}}{\sqrt{{{X}_{i}} QX_{i}^{T}}\sqrt{{{X}_{j}}QX_{j}^{T}}}~ \end{aligned}$$

where *Q* is the adjustment matrix:

19 $$\begin{aligned} Q=({{k}_{ij}}),{{k}_{i}}_{j}=\frac{\left| {{A}_{i}}\cap {{A}_{j}} \right| }{\left| {{A}_{i}}\cup {{A}_{j}} \right| } \end{aligned}$$

$$ {{A}_{i}}$$, $${{A}_{j}} $$ are the focal elements.

### BPA generation and combination method

The steps of BPA generation and combination are as follows.

**Step 1:** BPA generation.

**Step 1.1:** Calculate the distance ${d(x_{i},\mu _{j})}$ between the sample to be identified $x_{i}$ and each known population with the mean of $\mu _{j}$ according to Eq. (16).

**Step 1.2:** Take the distance ${d(x_{i},\mu _{j})}$ as $D(A_{i},B_{j})$ and calculate the similarity $S\left( A_{i},B_{j} \right)$ between the sample to be identified $A_{i} (x_{i})$ and each known population $B_{j} \mu _{i}$ according to Eq. (17).

**Step 1.3:** Normalize the similarity $S\left( A_{i},B_{j} \right)$ to obtain BPA.

**Step 2:** BPA combination.

**Step 2.1:** Calculate the similarity between the evidence vectors. With the independent evidence sources ${{m}_{i}}(i=1,2,\ldots ,l)$, corresponding evidence vectors are marked as ${{X}_{i}}(i=1,2,\ldots ,l)$, and the similarity ${s}_{ij}$ between ${{X}_{i}}$ and ${{X}_{j}} (i,j=1,2,\ldots ,l)$ can be calculated according to the Eq. (18).

**Step 2.2:** Generate the similarity matrix $M = ({{s}_{ij}})$.

**Step 2.3:** Calculate each similarity weight ${{\omega }_{i}}$ by the following formula:

20 $$\begin{aligned} {{\omega }_{i}}=\frac{\sum \nolimits _{j\ne i}{{{s}_{ij}}}}{\sum \nolimits _{i}{\sum \nolimits _{j\ne i}{{{s}_{ij}}}}} \end{aligned}$$

**Step 2.4:** Update the BPA. The method is shown in Algorithm 1.

**Step 2.5:** Calculate the standard BPA ${{m}_{std}}$ by the entropy weight method. The belief entropy ${{E}_{i}}$ of each mass function can be calculated by Eq. (15). Then the entropy weight for each BPA can be defined as:

21 $$\begin{aligned} {{\theta }_{i}}=\frac{1-{{e}_{i}}}{\sum \nolimits _{k=1}^{l} {(1-{{e}_{i}})}},{{e}_{i}}=\frac{{{E}_{i}}}{2|\Theta |{{\log }_{2}}({{2}^{|\Theta |}}-1)} \end{aligned}$$

The standard BPA can be calculated as follows:

22 $$\begin{aligned} {{m}_{\text {std}}}({{A}_{j}})=\sum \limits _{k=1}^{l}{{{\theta }_{k}}{{m}_{k}}({{A}_{j}})} \end{aligned}$$

**Step 2.6**: Fuse the mass functions including the standard BPA to obtain the final BPA according to Eq. (6):

23 $$\begin{aligned} m={{m}_{1}}\oplus {{m}_{2}}\oplus \cdots \oplus {{m}_{l}}\oplus {{m}_{std}} \end{aligned}$$

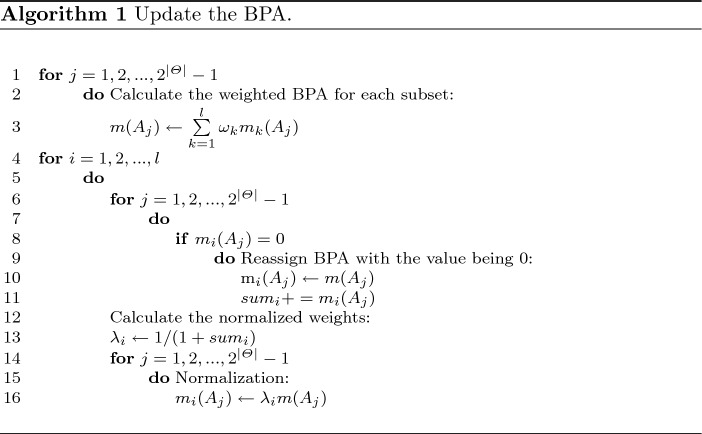


## Numerical examples

**Figure 1:**
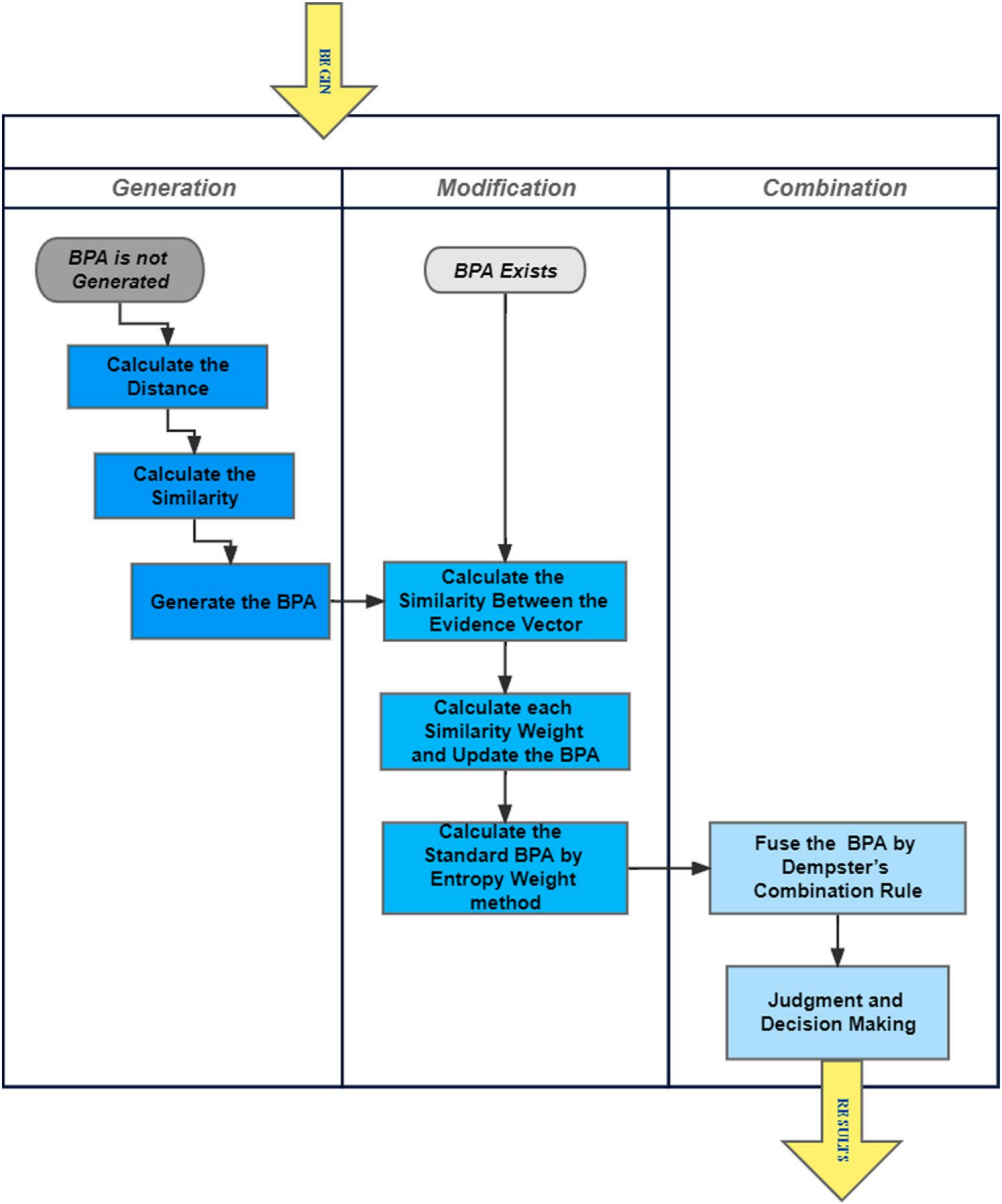
Flow chart for the proposed method.

The steps to use the proposed BPA generation and combination method are shown in Fig. 1. When the original BPAs are unknown, BPAs are generated by the proposed method. Then, modified BPAs and standard BPA can be fused by the proposed method. and judgment and decision can be made according to the final BPA. While when the original BPA is known, modification fusion of BPA is performed.

### An illustrative example

#### Example 1

Assume that a source reported by four information sources (e.g., sensors) of one sample are identified as (7.6, 3.0, 6.6, 2.1). This sample belongs to one of three categories, so the frame of discernment can be marked as $\{A, B, C\}$. The means and variances of each category can be calculated based on the four information sources. Table 1 shows the means and variances of category *A*.

According to the proposed method, the BPAs of the sample to be identified can be generated and fused based on the following steps to determine the category of the sample.

**Table 1:** The means and variances of category *A*.

Source	Mean	Variances
Sensor1	4.9878	0.1240
Sensor2	3.4317	0.1563
Sensor3	1.4512	0.0313
Sensor4	0.2390	0.0092

**Step 1.1:** Calculate the distance between the sample and each focal element in the frame of discernment, the results are in Table 2:

**Table 2:** The distance between the sample and each focal element.

	*A*	*B*	*C*	*AB*	*AC*	*BC*	*ABC*
Sensor1	55.0298	9.5246	2.8824	10.4735	4.0663	4.4165	4.8214
Sensor2	1.1923	0.4529	0.0047	0.0487	0.2595	0.1388	0.0229
Sensor3	847.5316	23.5018	4.5138	6.4912	2.4583	4.9291	2.8039
Sensor4	376.0782	15.1941	0.0483	5.1119	1.1933	1.0541	1.4528

**Step 1.2:** Calculate the similarity between the sample and each focal element in the frame of discernment, the results are in Table 3:

**Table 3:** The similarity between the sample and each focal element.

	*A*	*B*	*C*	*AB*	*AC*	*BC*	*ABC*
Sensor1	0.0133	0.0314	0.0556	0.0300	0.0472	0.0454	0.0436
Sensor2	0.0839	0.1294	0.5940	0.3119	0.1641	0.2116	0.3979
Sensor3	0.0034	0.0202	0.045	0.0378	0.0600	0.0431	0.0564
Sensor4	0.0051	0.0250	0.3128	0.0424	0.0839	0.0888	0.0766

**Step 1.3:** Calculate the BPA of the sample on each focal element in the frame of discernment, the results are in Table 4:

**Table 4:** The generated BPAs.

	*A*	*B*	*C*	*AB*	*AC*	*BC*	*ABC*
$BPA_{1}$	0.0499	0.1178	0.2087	0.1125	0.1773	0.1704	0.1634
$BPA_{2}$	0.0443	0.0684	0.3138	0.1648	0.0867	0.1118	0.2102
$BPA_{3}$	0.0129	0.0760	0.1691	0.1421	0.2256	0.1622	0.2120
$BPA_{4}$	0.0081	0.0394	0.4929	0.0668	0.1322	0.1399	0.1207

The original BPAs do not need to be adjusted. Jump to Step 2.5.

**Step 2.5:** Calculate the standard BPA according to the entropy weight method:

$$BPA_{std}=(0.1220, 0.1373, 0.1578, 0.1028, 0.1743, 0.1928, 0.1150)$$

**Step 2.6**: Fuse the BPAs including the standard BPA to obtain the final BPA:

$$BPA=(0.07407, 0.1163, 0.7849, 0.0050, 0.0112, 0.0111, 0.0004)$$

Therefore, it can be judged that the sample to be identified is most likely to belong to category *C*.

### BPA combination on data with high conflict

The proposed method is useful in solving the classical paradoxes. Some numerical examples are as follows, among which the detailed calculation process for Example 2 is shown.

#### Example 2

The frame of discernment is $\{a, b, c\}$, and the mass functions are shown in Table 5^51^.

When the Dempster’s combination rule is used, the outcomes are as follows:

$$\begin{matrix} m(a)=0.5 &{} {} &{} m(ab)=0.3 &{} {} &{} m(ac)=0.2 \\ m(b)=0 &{} {} &{} m(c)=0 &{} {} &{} m(abc)=0 \\ \end{matrix}$$

Since only the first evidence is presented, the BPA combination loses its meaning. The calculation process according to the method in this paper is as follows:

**Table 5:** Data of Example 2.

	*a*	*ab*	*ac*	*b*	*c*	*abc*
${m}_{1}$	0.5	0.3	0.2	0	0	0
${m}_{2}$	0	0	0	0.6	0.3	0.1
${m}_{3}$	0	0	0	0.5	0.3	0.2

**Step 2.1:** According to Eq. (19), the adjustment matrix can be calculated.

$$Q=\left[ \begin{matrix} 1 &{} \text {1/2} &{} \text {1/2} &{} 0 &{} 0 &{} 1/3 \\ \text {1/2} &{} 1 &{} 1/3 &{} \text {1/2} &{} 0 &{} 2/3 \\ 1/2 &{} 1/3 &{} 1 &{} 0 &{} \text {1/2} &{} 2/3 \\ 0 &{} \text {1/2} &{} 0 &{} 1 &{} 0 &{} 1/3 \\ 0 &{} 0 &{} \text {1/2} &{} 0 &{} 1 &{} 1/3 \\ 1/3 &{} 2/3 &{} 2/3 &{} 1/3 &{} 1/3 &{} 1 \\ \end{matrix} \right] $$

**Step 2.2:** The evidence similarity matrix can be calculated according to Eq. (18).

$$M=\left[ \begin{matrix} 1.0000 &{} 0.2880 &{} 0.3590 \\ 0.2880 &{} 1.0000 &{} 0.9873 \\ 0.3590 &{} 0.9873 &{} 1.0000 \\ \end{matrix} \right] $$

**Step 2.3:** According to Eq. (20), the weight of each evidence can be calculated, and the results are as follows:

$$\begin{aligned} &{{\omega }_{1}}=0.1979\text { }, {{\omega }_{2}}=0.3902, {{\omega }_{3}}=0.4119 \end{aligned} $$

**Step 2.4:** After the updates of zero BPAs and the normalization processing, the new mass functions are shown in Table 6.

**Table 6:** Results of Example 2.

	*a*	*ab*	*ac*	*b*	*c*	*abc*
${m}_{1}$	0.2775	0.1665	0.1110	0.2442	0.1335	0.0674
${m}_{2}$	0.0826	0.0496	0.0330	0.5009	0.2504	0.0835
${m}_{3}$	0.0826	0.0496	0.0330	0.4174	0.2504	0.1670

**Step 2.5:** The belief entropy weights of the new mass functions can be obtained according to Eq. (15):

$$\begin{aligned} &{{\theta }_{1}}=0.3219\text { } , {{\theta }_{2}}=0.3449 , {{\theta }_{3}}=0.3332 \end{aligned} $$

And the standard mass functions are as follows:

$$\begin{aligned} &{{m}_{std}}(a)=0.1416,\text { }{{m}_{std}}(ab)=0.0850 \\&{{m}_{std}}(ac)=0.0567,{{m}_{std}}(b)=0.3943 \\&{{m}_{std}}(c)=0.2150,\text { }{{m}_{std}}(abc)=0.1074 \\ \end{aligned} $$

**Step 2.6:** The Dempster’s combination rule is used to fuse ${{m}_{1}},{{m}_{2}},{{m}_{3}},{{m}_{std}}$, and the final mass functions are:

$$\begin{aligned} &m(a)=0.1209,\text { }m(ab)=0.0081 \\&m(ac)=0.0039,m(b)=0.7400 \\&m(c)=0.1264,\text { }m(abc)=0.0007 \\ \end{aligned} $$

This result is well interpretable for the original data.

#### Example 3

The frame of discernment is $\{A, T, C\}$, and the mass functions are as follows:

$$\begin{aligned} &{{m}_{1}}(A)=0.99,\text { }{{m}_{1}}(T)=0.01,\text { }{{m}_{1}}(C)=0 \\&{{m}_{2}}(A)=0,\text { }{{m}_{2}}(T)=0.01,\text { }{{m}_{2}}(C)=0.99 \\ \end{aligned} $$

When the Dempster’s combination rule is used, the outcomes are as follows:

$$\begin{aligned} m(A)=0,\text { }m(T)=1,\text { }m(C)=0 \end{aligned}$$

Obviously, the consequence goes against intuition. After using the method proposed in this paper, the final outcomes are as follows:

$$\begin{aligned} m(A)=0.5000,\text { }m(T)=0.0000,\text { }m(C)=0.5000 \end{aligned}$$

It can be seen that the final results correct the fusion paradox.

#### Example 4

The frame of discernment is $\{A, B, C\}$, and the mass functions are in Table 7.

**Table 7:** Data of Example 4.

	${m}_{1}$	${m}_{2}$	${m}_{3}$
A	0.98	0	0.9
B	0.01	0.01	0
C	0.01	0.99	0.1

The results with the Dempster’s combination rule are:

$$\begin{aligned} m(A)=0,\text { }m(B)=0,\text { }m(C)=1 \end{aligned}$$

By using the method in this paper, the final results are consistent with intuition:

$$\begin{aligned} m(\text {A})=0.9997,\text { }m(B)=0.0000,\text { }m(C)=0.0003 \end{aligned}$$

#### Example 5

The frame of discernment is $\{A, B, C\}$, and the mass functions are as follows:

$$\begin{matrix} {{m}_{1}}(A)=0.5 &{} {} &{} {{m}_{1}}(B)=0.2 &{} {} &{} \text { }{{m}_{1}}(C)=0.3 \\ {{m}_{2}}(A)=0 &{} {} &{} {{m}_{2}}(B)=0.9 &{} {} &{} {{m}_{2}}(C)=0.1 \\ {{m}_{3}}(A)=0.55 &{} {} &{} {{m}_{3}}(B)=0.1 &{} {} &{} {{m}_{3}}(C)=0.35 \\ {{m}_{4}}(A)=0.55 &{} {} &{} {{m}_{4}}(B)=0.1 &{} {} &{} {{m}_{4}}(C)=0.35 \\ {{m}_{5}}(A)=0.55 &{} {} &{} {{m}_{5}}(B)=0.1 &{} {} &{} {{m}_{5}}(C)=0.35 \\ \end{matrix}$$

The results obtained by the proposed method are compared with Dempster’s, Murphy’s and Deng’s methods, as shown in Table 8.

**Table 8:** Comparison of different methods of Example 5.

Method	*m*(*A*)	*m*(*B*)	*m*(*C*)
Dempster^6^	0	0.1228	0.8772
Murphy^49^	0.7958	0.0932	0.1110
Deng^35^	0.8909	0.0086	0.1005
Proposed method	0.9808	0.0019	0.0173

It is noticed that the new approach is more supportive for the target (focal element A) with the BPA being 0.9808.

### BPA combination on data without high conflict

The combination method in the proposed method not only performs well on highly conflicting data, but also shows superiority on the data without high conflict. Some numerical examples are as follow.

#### Example 6

The frame of discernment is $\{A, B\}$, and the mass functions are as follows:

$$\begin{matrix} {{m}_{1}}(A)=0.2 &{} {} &{} {{m}_{1}}(B)=0.3 &{} {} &{} \text { }{{m}_{1}}(AB)=0.5 \\ {{m}_{2}}(A)=0.5 &{} {} &{} {{m}_{2}}(B)=0.3 &{} {} &{} {{m}_{2}}(AB)=0.2 \\ \end{matrix}$$

The results with the Dempster’s combination rule are:

$$\begin{aligned} m(A)=0.4937, m(B)=0.3797, m(AB)=0.1266 \end{aligned}$$

The belief entropy of this group of BPAs can be calculated as 1.2394. By using the method proposed in this paper, the final results are as follows:

$$\begin{aligned} m(\text {A})=0.5425,\text { }m(B)=0.3959,\text { }m(AB)=0.0615 \end{aligned}$$

The belief entropy of the modified group of BPAs is 1.0417. By using the proposed method, the belief entropy decreases, which means the reduction in uncertainty of the judgment. In addition, the results of the proposed method provide more explicit support for A, while the support for the other two focal elements declines, which facilitates the judgment and decision making.

#### Example 7

The frame of discernment is $\{A, B, C\}$, and the mass functions are shown in Table 9.

**Table 9:** The data of Example 7.

	*A*	*B*	*C*	*AB*	*AC*	*ABC*
${m}_{1}$	0.3	0.3	0.2	0.1	0.1	0
${m}_{2}$	0	0.1	0.1	0.2	0.3	0.3

It can be calculated that the belief entropys of the original BPAs are 2.6520 and 3.0517, respectively. The results with the Dempster’s combination rule are as follows:

$$\begin{matrix} m(A)=0.3924, m(B)=0.2076, m(C)=0.1671 \\ m(AB)=0.0810, m(AC)=0.1063, m(ABC)=0.0456\\ \end{matrix}$$

The belief entropy of this group of BPAs can be calculated as 2.2984, which is close to that of the original BPAs. The uncertainty is still large, which is not conducive to further judgment. By using the method in this paper, the final results are as follows:

$$\begin{matrix} m(A)=0.3046, m(B)=0.3692, m(C)=0.2269 \\ m(AB)=0.0390, m(AC)=0.0390, m(ABC)=0.0153\\ \end{matrix}$$

The belief entropy of the modified group of BPAs is 1.7905, and the BPAs on focal elements AB, AC and ABC decrease. This result significantly reduces the belief entropy and enhances the certainty. In addition, the results with the Dempster’s combination rule are more supportive for focal element A, while the results of the proposed method support focal element B more.Compared with the original data with $${{m}_{1}}(A)=0.3, {{m}_{1}}(B)=0.3$$ and $${{m}_{2}}(A)=0, {{m}_{2}}(B)=0.1 $$, the result of the proposed method is more credible.

### Convergence verification

#### Example 8

The frame of discernment is $\{A, B, C\}$, and the mass functions are shown in Table 10.

The BPAs obtained by the method proposed are compared with that obtained by Dempster’s, Yager’s and Murphy’s methods. The results are shown in Table 11.

**Table 10:** The data of Example 8.

	*A*	*B*	*C*	*AB*	*AC*	*ABC*
${m}_{1}$	0.8	0.1	0	0	0	0.1
${m}_{2}$	0.4	0.2	0.1	0.3	0	0
${m}_{3}$	0	0.9	0.1	0	0	0
${m}_{4}$	0.5	0.2	0.05	0.2	0	0.05
${m}_{5}$	0.45	0.1	0	0	0.15	0.3

**Table 11:** The comparison of fusion BPA of Example 8.

	$m_{12}$	$m_{123}$	$m_{1234}$	$m_{12345}$
Dempster	m(A) = 0.8451 m(B) = 0.0986 m(C) = 0.00140 m(AB) = 0.0423	m(B) = 0.9890 m(C) = 0.0110	m(B) = 0.9975 m(C) = 0.0025	m(B) = 0.9980 m(C) = 0.0020
Yager^29^	m(A) = 0.600 m(B) = 0.0700 m(C) = 0.0100 m(AB) = 0.0300 m(ABC) = 0.2900	m(B) = 0.3605 m(C) = 0.0150 m(AB) = 0.0113 (ABC) = 0.6132	m(A) = 0.1944 m(B) = 0.2150 m(C) = 0.1381 m(AB) = 0.1229 m(ABC) = 0.2396	m(A) = 0.3528 m(B) = 0.2019 m(C) = 0.0812 m(AB) = 0.0369 m(AC) = 0.0258 m(ABC) = 0.3014
Murphy^49^	m(A) = 0.8219 m(B) = 0.1130 m(C) = 0.0103 m(AB) = 0.0514 m(ABC) = 0.0001	m(A) = 0.4615 m(B) = 0.5293 m(C) = 0.0017 m(AB) = 0.0074 m(ABC) = 0.0001	m(A) = 0.5342 m(B) = 0.4637 m(C) = 0.002 m(AB) = 0.0018 m(ABC) = 0.0001	m(A) = 0.6246 m(B) = 0.3661 m(C) = 0.0053 m(AB) = 0.0034 m(AC) = 0.0004 m(ABC) = 0.0002
Proposed method	m(A) = 0.7985 m(B) = 0.1279 m(C) = 0.0122 m(AB) = 0.0470 m(AC) = 0.0014 m(ABC) = 0.0129	m(A) = 0.7271 m(B) = 0.2603 m(C) = 0.0025 m(AB) = 0.0081 m(AC) = 0.0001 m(ABC) = 0.0018	m(A) = 0.8149 m(B) = 0.1820 m(C) = 0.0003 m(AB) = 0.0026 m(AC) = 0.0000 m(ABC) = 0.0001	m(A) = 0.8720 m(B) = 0.1273 m(C) = 0.0001 m(AB) = 0.0006 (AC) = 0.0000 (ABC) = 0.0001

According to Table 11, there is high conflict between $m_{3}$ and other mass functions. With Dempster’s combination rule, the outcomes of the fusion including $m_{3}$ assign a high value to focal element B, which is inconsistent with the original data. When the method proposed in this paper is used to fuse BPAs, due to the high value of the third BPA on focal element B, the support for A after fusing m1, m2 and m3 is lower than that with the fusion of m1 and m2, while the support degree of B is the opposite. With the addition of BPAs supporting focal element A, the fusion BPA on A gradually increased. Besides, the BPAs of AB, AC and ABC decreased with the increase of fusion times, which means the reduction of uncertainty. It can be seen that the fusion results of the method proposed are consistent with the original BPA, and compared with Yager’s and Murphy’s methods, the fusion BPA can quickly converge to the focal element as the number of BPAs supporting that focal element involved in fusion increases.

#### Example 9

The frame of discernment is $\{A, B, C\}$, and the mass functions are as follows:

$${{m}_{1}}(A)=0.7,{{m}_{1}}(AB)=0.3, {{m}_{i}}(C)=0.6,{{m}_{i}}(ABC)=0.4,i=2,3,4,5. $$

The fusion results of Dempster’s combination rule and the proposed method are shown Table 12.

**Figure 2:**
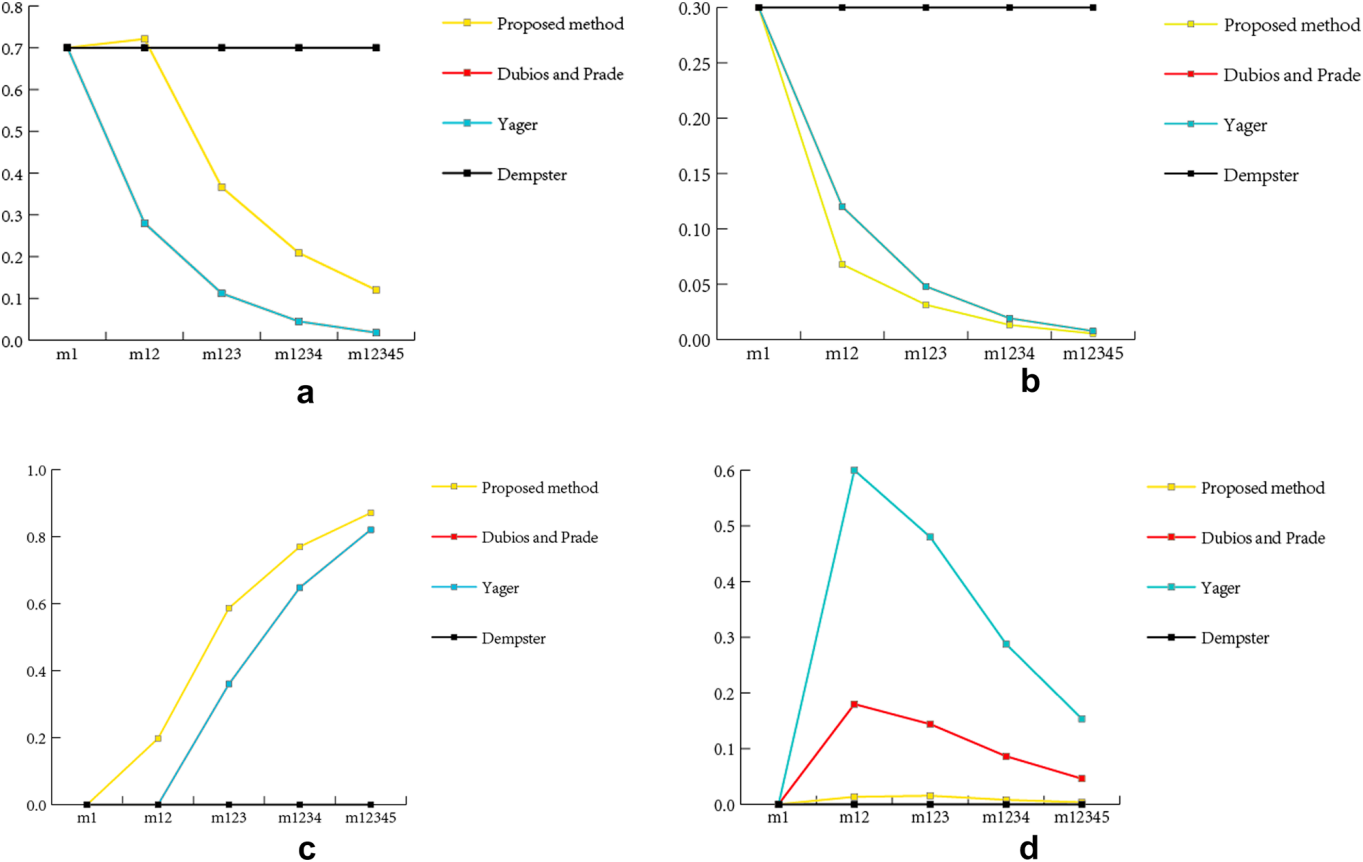
The changes of the fusion BPAs.

The results obtained by Dempster’s combination rule only reflect the value of $m_{1}$, while the fusion results of the method proposed gradually converge to focal element *A*. Figure 2 reflects the changes of the fusion BPAs on four focal elements combined by two methods with the increase of fusion times.

**Table 12:** The comparison of fusion BPA of Example 9.

	$m_{12}$	$m_{123}$	$m_{1234}$	$m_{12345}$
Dempster	m(A) = 0.7 m(AB) = 0.3	m(A) = 0.7 m(AB) = 0.3	m(A) = 0.7 m(AB) = 0.3	m(A) = 0.7 m(AB) = 0.3
Yager	m(A) = 0.28 m(AB) = 0.12 m(ABC) = 0.6	m(A) = 0.112 m(AB) = 0.048 m(C) = 0.36 m(ABC) = 0.48	m(A) = 0.0448 m(AB) = 0.0192 m(C) = 0.648 m(ABC) = 0.288	m(A) = 0.01792 m(AB) = 0.00768 m(C) = 0.8208 m(ABC) = 0.1536
Dubois and Prade	m(A) = 0.28 m(AB) = 0.12 m(AC) = 0.42 m(ABC) = 0.18	m(A) = 0.112 m(AB) = 0.048 m(C) = 0.36 m(AC) = 0.336 m(ABC) = 0.144	m(A) = 0.0448 m(AB) = 0.0192 m(C) = 0.648 m(AC) = 0.2016 m(ABC) = 0.0864	m(A) = 0.01792 m(AB) = 0.00768 m(C) = 0.8208 m(AC) = 0.10752 m(ABC) = 0.04608
Proposed method	m(A) = 0.7211 m(AB) = 0.0679 m(C) = 0.1975 m(ABC) = 0.0135	m(A) = 0.3663 m(AB) = 0.0314 m(C) = 0.5868 m(ABC) = 0.0154	m(A) = 0.2087 m(AB) = 0.0132 m(C) = 0.7701 m(ABC) = 0.0080	m(A) = 0.1201 m(AB) = 0.0054 m(C) = 0.8709 m(ABC) = 0.0036

In other methods used, it can be noticed that with the addition of $m_{i},i=2,3,4,5$, the support degree of fusion BPA calculated by three methods for focus elements A and AB gradually decreased, while the support degree for C gradually approached 1. But the fusion BPA calculated by proposed method can converge to the focal element more quickly than other two methods. In addition, the fusion BPA of ABC increased first but then decreased and tended to 0. This was because the high value of $m_{i}, i = 2,3,4,5$ on ABC provide more support for ABC. However, the increase of the BPA on ABC would increase the uncertainty of the results. The belief entropy of the fusion BPA would gradually decrease when fusing with the method proposed. Therefore, even if the fusion BPA of ABC increased for a short time, it would tend to 0 with the increase of fusion times. However, the variation of bps calculated by the other two methods is much larger than that by the proposed method. As a consequence, compared with Yager’s and Dubios and Prade’s, we can see that the BPA calculated by the new method is more stable.

## Application

All the following experiments and calculations are done in matlab software with C program language. The hardware for computation has the following parameters: Intel Core i7-8700, the CPU is 3.20 GHz, and with a 16 G RAM.

### The classification of Iris

#### Classify Iris by the proposed method

**Figure 3:**
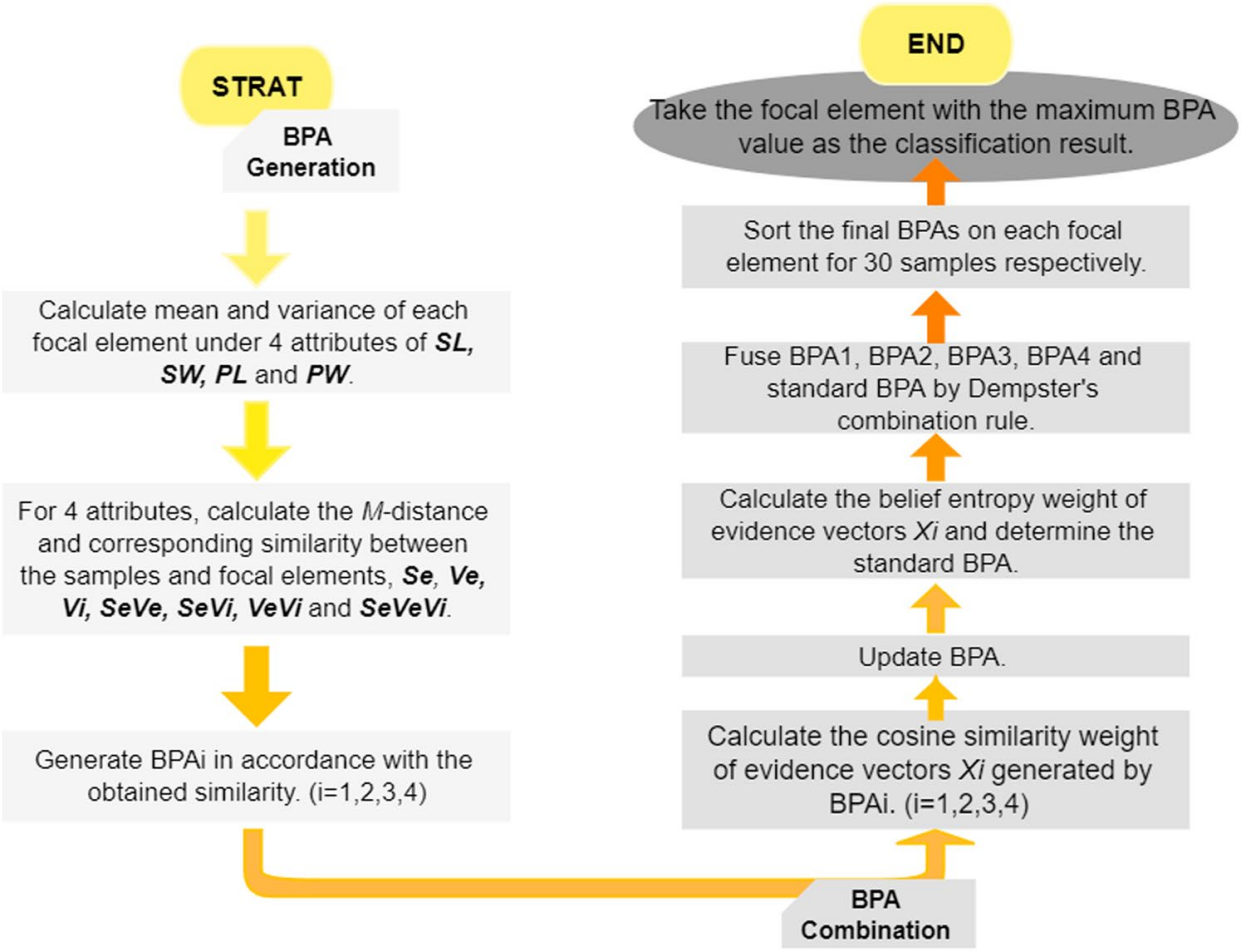
Prediction process on iris sets.

The method was used in the identification of iris. The test set and training set were divided according to 8:2, in which 120 data were taken as known datasets, and the remaining 30 data were samples to be identified. The three categories of iris were used to generated the frame of discernment, which can be expressed as $\{Setosa (Se), Versicolor (Ve), Virginica (Vi)\}$. The four attributes of iris, *SL*, *SW*, *PL*, *PW*, can be regarded as four sensors. The experiment process is shown in Fig. 3.

**Figure 4:**
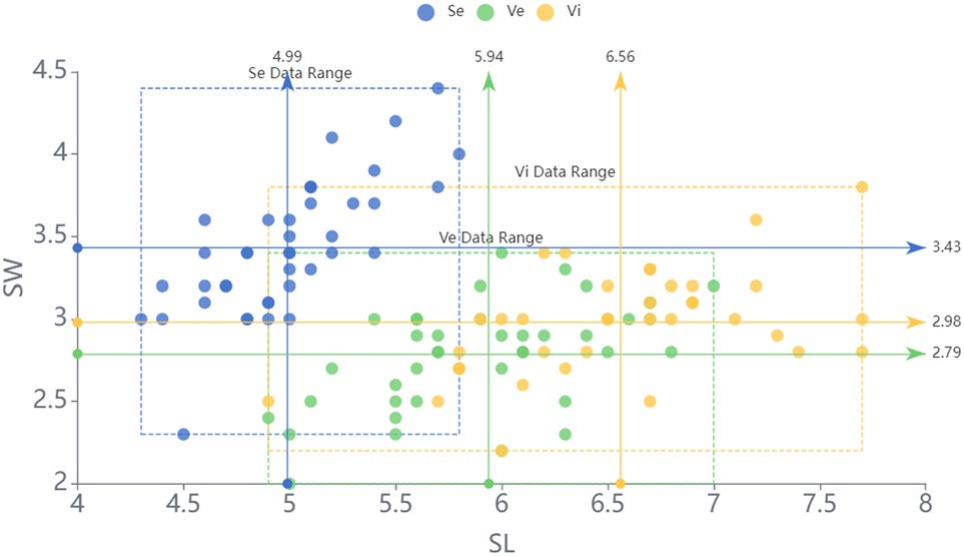
The distribution of iris data.

Figure 4 shows the training data distribution on attributes *SL* and *SW*, and the data range and mean of each category *Se, Ve, Vi* are labeled in the graph. The mean and variance of each focal element can be estimated by the sample distribution. It can be noticed that the data of *Se* shows obvious differences from that of *Ve* and *Vi* on the attribute *SL* and *SW*, while the data distributions of *Ve* and *Vi* overlap to a certain degree.

**Figure 5:**
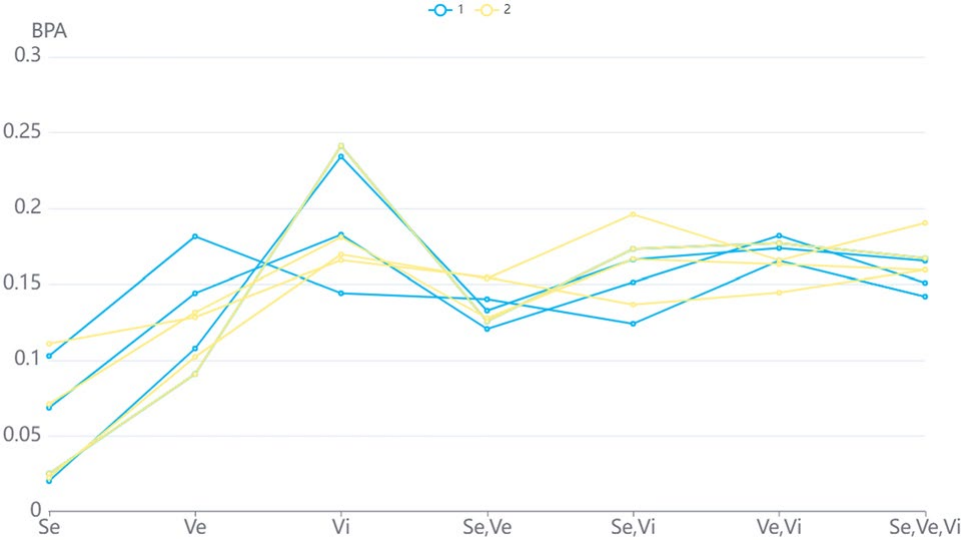
The generated BPAs of the first two samples.

The BPAs of samples to be identified on each attribute generated in accordance with the proposed method are shown in Fig. 5. For observation purposes, only the BPAs of the first two samples are displayed. Blue and Yellow lines represent the BPAs on each focal element of the first two samples respectively. It can be seen that there is no significant conflict between BPAs of the sample data. For categories *Ve* and *Vi*, BPAs are close, and the proposed fusion method was used for further calculation and judgment.

**Figure 6:**
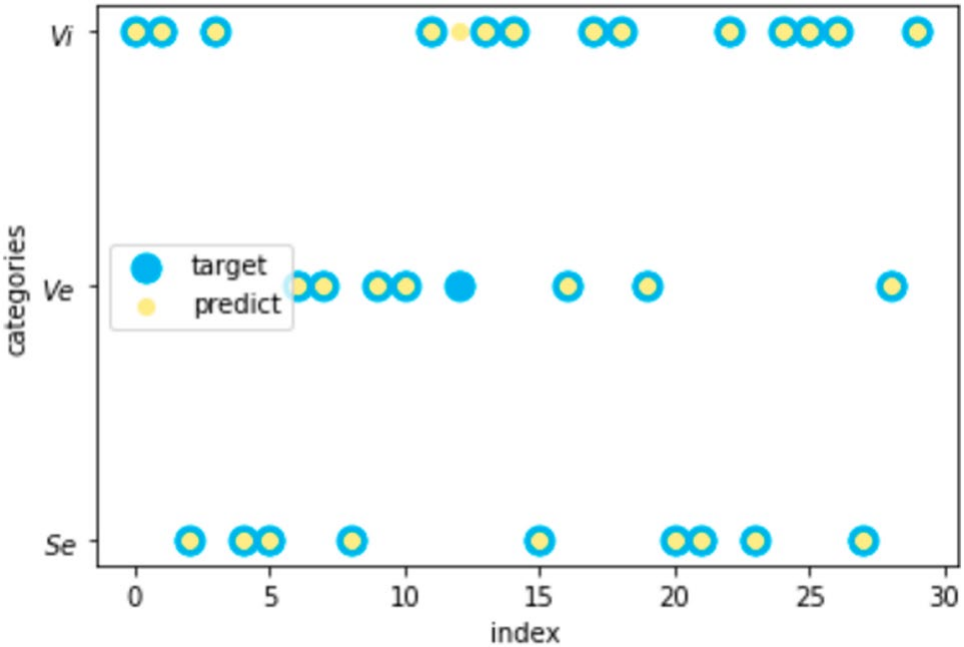
The predicted results on iris data with the proposed method.

BPAs were modified according to the above steps, and the standard BPAs of each sample to be identified were obtained. The BPAs on the four attributes and the standard BPA were fused, and the final BPA was used to classify the iris. The predicted results on the test set were calculated, and the prediction accuracy of all the samples to be identified is 96.7%. The prediction outcomes are shown in Fig. 6.

Where yellow points are the prediction results, and blue points refer to the real category. Only one sample was misclassified. Check the BPA for misclassified sample. It can be found that the BPA for Ve, 0.3770, is slightly less than the BPA for Vi, 0.4143.

### Comparison with the Dempster’s combination rule

The accuracy obtained by the Dempster’s combination rule is also 96.7%, and the misclassified sample is the same as that with the proposed method. Compare the BPAs obtained by Dempster’s combination rule and the proposed method. Table 13 shows BPAs of the first six samples.

**Table 13:** Comparison of BPAs with the two methods.

Index	Method	Se	Ve	Vi	SeVe	SeVi	VeVi	SeVeVi
1	Dempster	0.1199	0.2814	0.5271	0.0165	0.0229	0.0307	0.0016
	New	0.1307	0.2733	0.5597	0.0076	0.0132	0.0150	0.0004
2	Dempster	0.1558	0.2496	0.5117	0.0213	0.0306	0.0289	0.0021
	New	0.1666	0.2485	0.5432	0.0097	0.0172	0.0145	0.0005
3	Dempster	0.5590	0.1844	0.1751	0.0340	0.0325	0.0129	0.0019
	New	0.5711	0.1975	0.1893	0.0175	0.0164	0.0067	0.0005
4	Dempster	0.1413	0.2764	0.5027	0.0195	0.0268	0.0316	0.0019
	New	0.1557	0.2698	0.5337	0.0092	0.0159	0.0154	0.0005
5	Dempster	0.6163	0.1598	0.1511	0.0299	0.0304	0.0108	0.0016
	New	0.6259	0.1782	0.1591	0.0157	0.0153	0.0056	0.0004
6	Dempster	0.5160	0.2090	0.1968	0.0307	0.0304	0.0153	0.0019
	New	0.5281	0.2256	0.2059	0.0166	0.0149	0.0078	0.0005

Take the first sample as an example. The target category is *Vi*, and the BPA of *Vi* obtained by Dempster’s combination rule and the proposed method are 0.5271 and 0.5597, respectively. Compare the rest of the samples in the same way, and it can be found that the BPA with the proposed method support the correct result more.

### Comparison with the K-nearest neighbors algorithm

**Figure 7:**
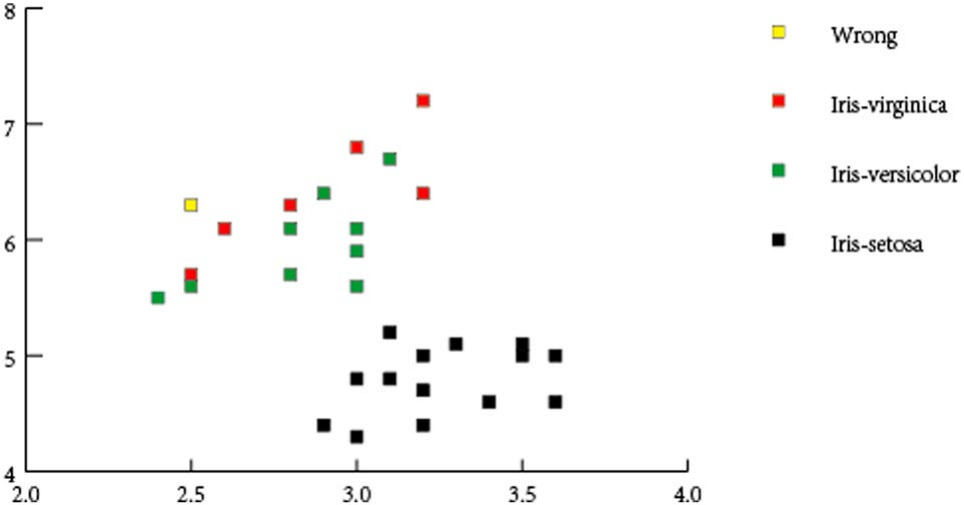
The predicted results on iris set by KNN algorithm.

The K-nearest neighbors (KNN) algorithm was used to classify Iris. We set the neighbors K = 5. After training the model, the test results are shown in Fig. 7. The horizontal axis represents Sepal Length and the vertical axis represents Sepal Width. We set 0 for Iris-setosa, 1 for Iris-versicolor and 2 for Iris-virginica. The Classification results of model prediction is:

{0 0 1 0 0 0 0 0 0 0 0 1 2 2 1 2 1 1 0 1 1 2 2 0 2 1 1 2 0 0 }.

The real classification results is:

{0 0 1 0 0 0 0 0 0 0 0 1 2 2 1 2 1 1 0 1 1 2 2 0 2 1 1 1 0 0}.

Thus, the accuracy rate of classfication is 96.7%.The proposed method achieves the same accuracy as the KNN algorithm. The accuracy of the proposed method is proved.

### Comparison with BP neural network classification method

**Figure 8:**
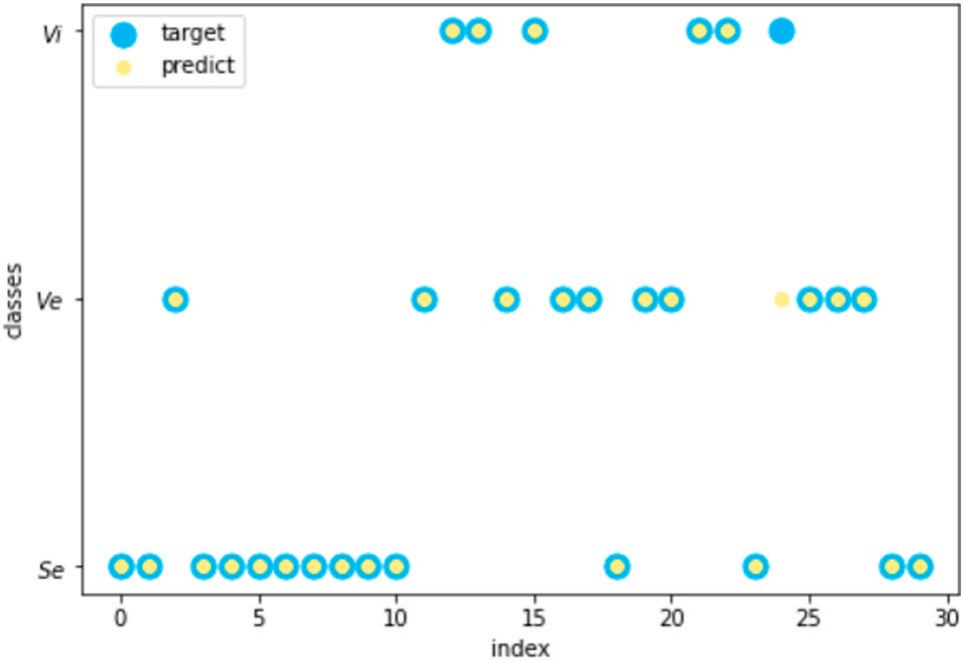
The predicted results on iris set by BP neural network.

The BP neural network with a single hidden layer was also used to classify iris. The parameters selection were as follows. There were three nodes in the hidden layer and the learning step was 0.1. After 300 iterations, the convergence rate of the loss function was very slow and the classification results tended to be stable. In this case, the accuracy rate of classification is 96.7%. The prediction results are shown in Fig. 8, where yellow points are the prediction results, and blue points refer to the real category.

The method proposed in this paper achieves the same accuracy as the BP neural network in the classification of iris, which also demonstrates that the generation and combination of BPA with the method in this paper is effective.

### The classification of wine

The method was used in the identification of wine. The test set and training set were divided according to 8:2, in which 1279 data were taken as known datasets, and the remaining 320 data were samples to be identified. These wines can be classified as good, normal and bad, and the frame of discernment can be expressed as $\{Bad (B), Normal (N), Good (G)\}$. The 11 attributes of wine can be regarded as 11 sensors.

**Figure 9:**
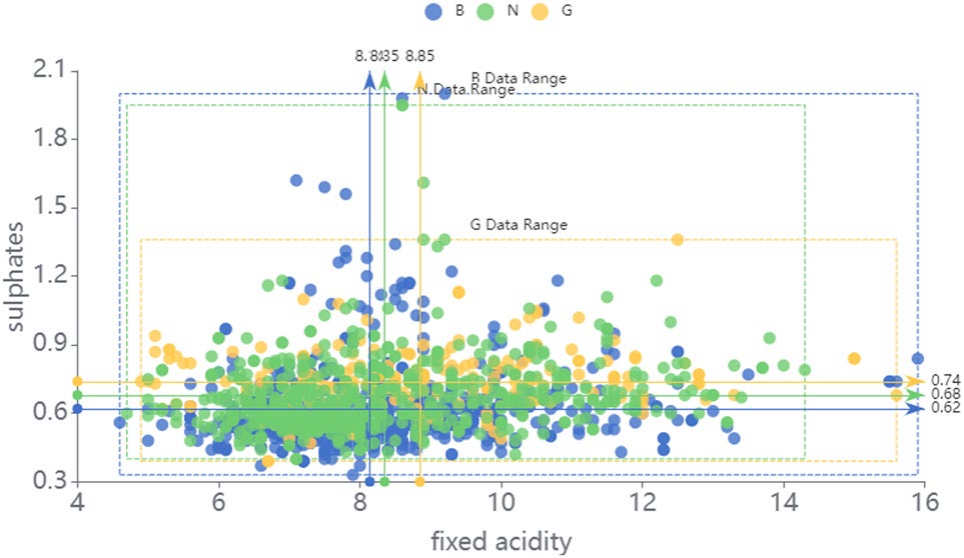
The distribution of wine data.

Figure 9 reflects the distribution of wine data on fixed acidity and sulphates, and the data range and mean of each category *B, N, G* are labeled in the graph. The mean and variance of each focal element can be estimated by the sample distribution. It can be noticed that the distribution of these three categories are similar, which adds to the difficulty of classification.

#### Comparation of the classification with the two methods, BP neural network and decision tree

The method proposed in this paper and the Dempster’s combination rule were used for BPA generation and fusion, and both the accuracy of classification are 58.13%.

The classification result of the sixth sample is wrong. Take it as an example to analyze the cause of the error. Sample 6 should be “Normal”, but both the results obtained by the two methods are “Good”. The BPAs for “Good” are 0.3509 and 0.3508 respectively, and the BPA for “Normal” are 0.3447 and 0.3447, respectively. There is no significant difference in confidence between the two types, which makes the judgment difficult. Table 14 shows the generated BPAs and the fusion BPA with two methods of the sixth sample. It can be observed that there are 7 original BPAs support “Good” more than “Normal”. Thus, the error of judgment in this case comes from the inconspicuous feature of the sample.

**Table 14:** The BPA of each sample.

	B	N	G	BN	BG	NG	BNG
Dempster	0.30404	0.34469	0.35078	0.00016	0.00016	0.00017	0
New method	0.30412	0.34471	0.35092	0.00008	0.00008	0.00009	0
BPA1	0.13816	0.14402	0.14794	0.14123	0.14113	0.14514	0.14238
BPA2	0.13808	0.14478	0.14523	0.14127	0.14137	0.14657	0.14270
BPA3	0.13818	0.14323	0.14885	0.14080	0.14166	0.14497	0.14231
BPA4	0.14300	0.14366	0.14130	0.14330	0.14263	0.14307	0.14304
BPA5	0.14168	0.14275	0.14529	0.14215	0.14233	0.14331	0.14250
BPA6	0.14213	0.14241	0.14477	0.14227	0.14275	0.14304	0.14263
BPA7	0.14055	0.14262	0.14686	0.14170	0.14202	0.14382	0.14242
BPA8	0.14219	0.14363	0.14165	0.14326	0.14281	0.14324	0.14324
BPA9	0.14259	0.14231	0.14417	0.14246	0.14297	0.14280	0.14270
BPA10	0.13992	0.14477	0.14096	0.14214	0.14243	0.14644	0.14334
BPA11	0.13218	0.14739	0.14221	0.14135	0.14213	0.15040	0.14434

The BP neural network with a single hidden layer was also used to classify wine. The parameters selection were as follows: there were six nodes in the hidden layer and the learning step was 0.1. After 500 iterations, the classification results tended to be stable. In this case, the accuracy rate of classification is 60.0%. When using the cart tree method in the decision tree for classification, the final accuracy is 57.19%. The KNN algorithm was also applied to the classification of wine. First we divide the test set and training set. Then we normalized the data to speed up the convergence of the training network and increase the accuracy. The final accuracy is 55.94%, which is lower than the accuracy calculated by the proposed method.

For the classification of wine, the accuracy obtained by the proposed method is higher than the results obtained by the KNN algorithm and decision tree. And it is close to The BP neural network with a single hidden layer. The proposed method has reached a relatively high accuracy in this problem, which also demonstrates the effectiveness of the proposed method.

## Conclusion

In this paper, a novel BPA generation and combination method was proposed. The evidence source was modified by reinterpreting the BPA with the value being 0, and the weighted standard BPA was obtained with consideration of both the reliability and uncertainty of the evidence. The results were well verified in the correction of various highly conflicting data fusion paradoxes, and the fusion results with the method proposed show higher degree of support for correct target than the existing classical methods for conflicting data fusion. Besides, compared with Dempster’s combination rule, the proposed method also reduces the belief entropy of the final BPA, which means the reduction of the uncertainty of the results, and is beneficial for decision making.

Additionally, experiments on the datasets demonstrate the rationality of the method. The public iris and wine datasets were used in the experiment. With BPA generation and combination method proposed in this paper, the classification accuracy of the iris is 96.7%, as high as the result from the BP neural network with single hidden layer. For the wine quality classification experiment, the accuracy with the proposed method is 58.13%, slightly lower than the result from the BP neural network with single hidden layer, but slightly higher than that from cart decision tree and KNN algorithm.

Some possible directions for the following work are as follows. Since the modification value of the zero BPA was obtained by taking a weighted average of all the evidence, the assignment is only an estimate in the average level. However, the real BPA provided by the sensors in the same condition is difficult to estimate. How to update BPA in a more reasonable way is still a question to be discussed.

## Data Availability

All data are included in the manuscript.
